# Induction of *Mycobacterium Tuberculosis* Lipid-Specific T Cell Responses by Pulmonary Delivery of Mycolic Acid-Loaded Polymeric Micellar Nanocarriers

**DOI:** 10.3389/fimmu.2018.02709

**Published:** 2018-11-27

**Authors:** Shaobin Shang, Dina Kats, Liang Cao, Eva Morgun, Diana Velluto, Ying He, Qichen Xu, Chyung-Ru Wang, Evan A. Scott

**Affiliations:** ^1^Department of Microbiology and Immunology, Northwestern University, Chicago, IL, United States; ^2^Interdisciplinary Biological Sciences Program, Northwestern University, Evanston, IL, United States; ^3^Diabetes Research Institute and Cell Transplant Center, University of Miami School of Medicine, Miami, FL, United States; ^4^Department of Biomedical Engineering, Northwestern University, Evanston, IL, United States; ^5^Simpson Querrey Institute, Northwestern University, Chicago, IL, United States; ^6^Chemistry of Life Processes Institute, Northwestern University, Evanston, IL, United States

**Keywords:** *Mycobacterium tuberculosis*, mycolic acid, T cells, CD1, lipid antigens, micelles, subunit vaccines

## Abstract

Mycolic acid (MA), a major lipid component of *Mycobacterium tuberculosis* (Mtb) cell wall, can be presented by the non-polymorphic antigen presenting molecule CD1b to T cells isolated from Mtb-infected individuals. These MA-specific CD1b-restricted T cells are cytotoxic, produce Th1 cytokines, and form memory populations, suggesting that MA can be explored as a potential subunit vaccine candidate for TB. However, the controlled elicitation of MA-specific T cell responses has been challenging due to difficulties in the targeted delivery of lipid antigens and a lack of suitable animal models. In this study, we generated MA-loaded micellar nanocarriers (MA-Mc) comprised of self-assembled poly(ethylene glycol)-bl-poly(propylene sulfide; PEG-PPS) copolymers conjugated to an acid sensitive fluorophore to enhance intracellular delivery of MA to phagocytic immune cells. Using humanized CD1 transgenic (hCD1Tg) mice, we found these nanobiomaterials to be endocytosed by bone marrow-derived dendritic cells (DCs) and localized to lysosomal compartments. Additionally, MA-Mc demonstrated superior efficacy over free MA in activating MA-specific TCR transgenic (DN1) T cells *in vitro*. Following intranasal immunization, MA-Mc were primarily taken up by alveolar macrophages and DCs in the lung and induced activation and proliferation of adoptively transferred DN1 T cells. Furthermore, intranasal immunization with MA-Mc induced MA-specific T cell responses in the lungs of hCD1Tg mice. Collectively, our data demonstrates that pulmonary delivery of MA *via* PEG-PPS micelles to DCs can elicit potent CD1b-restricted T cell responses both *in vitro* and *in vivo* and MA-Mc could be explored as subunit vaccines against Mtb infection.

## Introduction

*Tuberculosis (TB)*, the disease caused by *Mycobacterium tuberculosis* (Mtb), remains one of the world's deadliest communicable diseases (1). The waxy cell wall of Mtb contains several unique lipids which are highly distinct from mammalian lipids and influence mycobacterial viability, making them attractive targets for immune defense. Indeed, several of lipids derived from the mycobacterial cell wall can be recognized by CD1-restricted T cells (2–7).

The CD1 family of antigen presenting molecules is specialized in presenting lipid/glycolipid antigens to T cells (6, 8). Humans express group 1 CD1 molecules CD1a, CD1b, and CD1c, and the group 2 molecule, CD1d. Mice, however, only express CD1d (8). Among four CD1 isoforms, CD1b presents the largest pool of Mtb-derived lipids, including mycolic acid (MA), the key structural element of Mtb's outer membrane (8, 9). MA broadly distributed within endosomal compartments of dendritic cells and MA-specific CD1b-restricted T cells can be detected in the blood (2) and disease sites of tuberculosis patients and demonstrated a memory response upon *ex vivo* re-stimulation (10). These MA-specific CD1b-restricted T cells are cytotoxic and produce proinflammatory cytokines IFN-γ and TNF-α, crucial for anti-Mtb immunity (2, 11, 12). In addition, adoptive transfer of MA-specific CD1b-restricted T cells confers protection to Mtb infection in a human group 1 CD1 transgenic (hCD1Tg) mouse model (13, 14). These data suggest that MA may be harnessed as components of novel vaccines against Mtb infection.

MA has very limited solubility and micellar stability in aqueous solutions, making efficient *in vivo* delivery a considerable challenge. Furthermore, presentation of MA requires complexation with CD1b molecules within lysosomes, which necessitates intracellular delivery (15). One strategy to address these issues is by packaging the lipid within a nanobiomaterial-based carrier with enhanced capability for *in vivo* endolysosomal delivery to antigen presenting cells (APCs), particularly dendritic cells (DCs). Such nanocarriers have become increasingly engineered and utilized for vaccination and immunotherapy to decrease non-specific cellular interactions, transport combinations of molecules with diverse physicochemical properties and enhance endocytosis by APC (16, 17).

Nanocarriers self-assembled from poly(ethylene glycol)-*bl*-poly(propylene sulfide; PEG-PPS) copolymers have demonstrated considerable utility for intracellular delivery of immunostimulants and antigens (18–23). PEG-PPS assembles into lyotropic mesophases, enhancing overall aggregate stability under a range of conditions (18, 24, 25). Even at relatively low MW, PEG-PPS nanobiomaterials are highly stable in dilute aqueous solutions (26). An advantageous characteristic of PEG-PPS is that the PPS block is oxidation-sensitive and converts to the progressively more water soluble poly(propylene sulfoxide) and subsequently poly(propylene sulfone) derivatives in the presence of physiologic levels of reactive oxygen species (ROS) (22, 25, 27). This allows efficient disassembly of nanocarriers within APC lysosomes as well as early and late endosomes for enhanced antigen presentation and adjuvant stimulation (19–22). PEG-PPS nanocarriers have therefore been extensively employed for endosomal and lysosomal delivery to APCs (18–22, 28), and possess physicochemical properties beneficial for the controlled delivery of MA.

To study the dynamics and *in vivo* function of group 1 CD1-restricted T cells during Mtb infection, we have generated human group 1 CD1 transgenic mice (hCD1Tg) that mimic the human expression of group 1 CD1 as well as a MA-specific TCR transgenic mouse strain (DN1Tg/hCD1Tg) (13, 14). In this study, we have synthesized, assembled, and employed MA-loaded PEG-PPS micellar nanocarriers to induce and characterize MA-specific T cell responses following pulmonary delivery in hCD1Tg mice. We synthesized two separate PEG-PPS fluorescent conjugates, each possessing the same copolymer but with distinct fluorophores to characterize MA delivery to and presentation by DCs both *in vitro* and *in vivo*. MA-loaded acid-sensitive fluorophore-conjugated micelles (MA-ASMc) (29) were employed to verify lysosomal delivery within bone marrow derived DCs (BMDCs) *in vitro* by confocal microscopy. Following intranasal administration, MA-ASMc additionally supported flow cytometric analysis of cellular biodistributions while MA-loaded micelles conjugated to a near-infrared fluorescence (NIRF) sensitive fluorophore (MA-NIMc) allowed assessment of the organ level biodistributions. By employing PEG-PPS nanobiomaterials with hCD1Tg mice, we present a versatile strategy that could be used to design and test future vaccine formulations that incorporate lipid antigens.

## Materials and methods

### Ethics statement

This study was carried out in accordance with the recommendations in the Guide for the Care and Use of Laboratory Animals of the National Institutes of Health. The protocol was approved by the Institutional Animal Care and Use Committee of the Northwestern University (Protocol number: IS00004890).

### Mice

Human CD1 transgenic mice (hCD1Tg) in B6 or MHC II-deficient background (14) and CD1b-restricted MA-specific TCR transgenic mice in Rag^−/−^ background (DN1Tg/hCD1Tg/Rag^−/−^) (13) were generated and maintained in house.

### Mtb lipid antigens and antibodies

MA (MW 1,100–1,300 Da) was purchased from Sigma-Aldrich (St. Louis, MO) and reconstituted in an organic solution that comprised of chloroform and methanol at a ratio of 3 to 1 and stored as aliquots at −20°C. Monoclonal antibodies against mouse CD11b (M1/70), CD11c (N418), NK1.1 (PK136), CD19 (6D5), Ly6G (1A8), CD25 (PC61), CD44 (1M7), CD69 (H1.2F3), CD103 (2E7), F4/80 (BM8), TCRβ (H57-597), Siglec F (E50-2440) and human TCR Vβ5.1 (LC4) with different fluorochrome conjugates were purchased either from BioLegend or eBioscience (San Diego, CA).

### Synthesis of fluorescent PEG_44_-PPS_15_ copolymers

An acid-sensitive fluorophore (ASF, λ_ex_ = 395 nm, λ_em_ = 505 nm) derived from a 1,8-naphthalamide was synthesized as previously described (29, 30). The fluorophore was then modified to introduce a -SH containing linker on the naphthalimide ring for conjugation to PEG-PPS (31) (Figure S1). The N-Quinolin-8-yl−4 bromo-1,8-naphthalimide was formed by mixing equal molar equivalents of 8-aminoquinoline and 4-bromo-1,8-napthalic anhydride in methanol and heating while stirring for two days. The mixture was cooled and the precipitate was collected by filtration. The product (**a**) was then reacted with 1.5 equivalents of mercaptoethanol and potassium carbonate in dimethylformamide (DMF) overnight to yield N-(quinolin-8-yl-4-mercaptoethanol)-1,8-napthalamide (**b**) that was isolated by precipitation into water followed by filtration. The hydroxyl group at the end of the linker was then modified by reaction with mesylate chloride in dichloromethane (DCM) in the presence of triethylamine overnight to generate N-quinolin-8-yl-napthalamide mesylate (**c**). The DCM was removed and the mesylate derivative was washed with water, dried and further reacted with thioacetic acid and potassium carbonate in DMF overnight. The product was isolated by precipitation in non-saturated NaCl to obtain the N-quinolin-8-yl-napthalamide thioacetate. The thio-protected group was used to initiate the ring opening polymerization of 15 molar equivalents of propylene sulfide in DMF for 1 h, before end-capping with aldrithiol-2 which provides a disulfide link at the end of the PPS chain, useful for further substitution via disulfide exchange. The resulting polypropylene sulfide chain (PPS) with acid sensitive fluorophore was purified by precipitation in cold methanol and mixed with sodium methoxide-activated PEG_44_ thioacetate (PEG_44_-TAA) that was generated as previously described (18), in DMF overnight. The disulfide exchange reaction yielded PEG_44_-PPS_15_-ASF. PEG-PPS block copolymer used for *in vivo* NIRF imaging experiments was synthesized as previously described to possess a terminal free thiol (23), which was subsequently reacted with maleimide functionalized DyLight 755 (ThermoFisher Scientific) after micellar assembly to form PEG_44_-PPS_15_-DyLight 755. All the products obtained were confirmed by ^1^HNMR (Bruker Avance III 500MHz):

*N-Quinolin-8-yl*−*4 bromo-1,8-naphthalimide* (**a**)
  ^1^H–NMR (400 MHz, CDCl_3_): δ 8.80 (1H, *dd*), 8.72 (1H *dd*), 8.66 (1H, *dd*), 8.48 (1H, *d*), 8.25 (1H, *dd*), 8.10 (1H, *d*), 7.99 (1H, *dd*), 7.90 (1H, *dd*), 7.75 (2H, *m*), 7.42 (1H, *dd*).
*Mercaptoethanol derivative* (**b**)
  ^1^H–NMR (400 MHz, CDCl_3_): δ 8.80 (1H, *dd*), 8.70 (2H, *m*), 8.54 (1H, *d*), 8.24 (1H, *dd*,), 7.98 (1H, *dd*), 7.82 (1H, *dd*), 7.78 (1H, *dd*), 7.71 (2H, *m*), 7.42 (1H, *dd*), 3.96 (2H, *dd*), 3.40 (1H, *t*), 2.04 (1H, *t*).
*Mesylate derivative* (**c**)
  ^1^H–NMR (400 MHz, CDCl_3_): δ 8.80 (1H, *dd*), 8.70 (2H, *m*), 8.60 (1H, *d*), 8.24 (1H, *dd*,), 7.98 (1H, *dd*), 7.82 (1H, *dd*), 7.75 (2H, *dd*), 7.67 (1H, *m*), 7.38 (1H, *dd*), 4.42 (2H, *t*), 3.50 (2H, *t*), 2.99 (3H, *s*).
*Thioacetate derivative* (**d**)
  ^1^H–NMR (400 MHz, CDCl_3_): δ 8.80 (1H, *dd*), 8.70 (2H, *m*), 8.60 (1H, *d*), 8.24 (1H, *dd*,), 7.98 (1H, *dd*), 7.82 (2H, *m*), 7.77 (1H, *dd*), 7.71 (1H, *m*), 7.42 (1H, *dd*), 3.38 (2H, *t*), 3.40 (2H, *t*), 2.40 (3H, *s*).
*PPS*_15_*-ASF* (**e**)
  ^1^H–NMR (400 MHz, CDCl_3_): δ 1.35–1.45 (d, C*H*3 in PPS chain), 2.6–2.7 (m, C*H* in PPS chain), 2.85–3.0 (m, C*H*_2_ in PPS chain), 7.8–7.83 (m, 1H, pyridine group).
*PEG-ss-PPS*_15_*-ASF* (**e**)
  ^1^H NMR (CDCl_3_): δ 1.35–1.45 (d, C*H*_3_ in PPS chain), 2.6–2.7 (m, C*H* in PPS chain), 2.85–3.0 (m, C*H*_2_ in PPS chain), 3.38 (s, 3H, -OC*H*_3_), 3.52–3.58 (t, 2H, -OC*H*_2_CH_2_S), 3.5–3.7 ppm (broad, PEG chain protons).

### Micelle nanocarrier formation and loading efficiency

Empty/vehicle acid sensitive micelles (V-ASMc) or MA-loaded micelles (MA-ASMc) were formed by dissolving 10 mg of PEG_44_-PPS_15_-ASF copolymer in 500 uL of chloroform, with ,or without 100 μg of MA, followed by the addition of 1 ml of endotoxin-free phosphate buffered saline (PBS). The mixture was stirred until chloroform was no longer present. V-ASMc and MA-ASMc were then centrifuged at 10,000 RPM for 5 min to remove precipitates. NIRF-sensitive PEG_44_-PPS_15_-DyLight 755 micelles with (MA-NIMc) and without loaded MA (V-NIMc) were formed in a similar manner, with Dylight 755 (Thermo Fischer Scientific) added after nanocarrier formation and allowed to mix overnight. Excess dye was removed by gravity filtration on a Sephadex LH-20 column (GE Healthcare Life Sciences). Resultant nanocarriers were characterized by cryo-transmission electron microscopy (cryoTEM) and dynamic light scattering (DLS). To test the loading efficiency, MA was labeled with 4-bromomethyl-6,7-dimethoxycoumarin (Sigma-Aldrich) at 90°C for 20 min in chloroform, with a molar excess of MA, then loaded into the copolymer to generate MA-loaded micelles as described above. Nanocarriers were then purified on an LH20 gravity column and the fluorescence of the derivatized MA was measured using a spectrophotometer (λ_ex_ = 365 nm, λ_em_ = 410 nm) (32).

### Cell preparation and flow cytometry

Single-cell suspensions were prepared from the lung, spleen, and mediastinal lymph nodes by mechanical disruption in HBSS/2% FBS. Lung was digested with collagenase IV (1 mg/ml; Sigma) and DNase I (30 μg/ml; Sigma) for 30 min at 37°C before disruption. For cell surface staining, cells were pre-incubated with 2.4G2 Fcγ RII/RIII blocking mAb for 15 min and then stained with the appropriate combinations of mAbs listed below in HBSS/2% FBS for 30 min at 4°C to define alveolar macrophages (SiglecF^+^CD11b^−^CD11c^+^), dendritic cells (CD11b^+^CD11c^+^), monocytes (CD11b^+^CD11c^−^), neutrophils (CD11b^+^Ly6G^+^), T cells (TCRβ^+^), B cells (B220^+^), and NK cells (NK1.1^+^TCRβ-) cells. DN1 T cells are human TCR Vβ5.1-positive. CD25, CD44, CD69, CD62L, CCR7, and CD103 were used to define T cell activation. For intracellular cytokine staining, the procedure was performed as previously described (14) and stained with anti-IL-2, IFN-γ, and TNF-α or isotype matched control antibodies. All mAbs were purchased form BioLegend (San Diego, CA) or BD Bioscience (San Jose, CA). Flow cytometry was performed with a FACS CantoII (BD Biosciences, San Jose, CA) and analyzed using FlowJo software (Tree Star, Ashland, OR).

### Dendritic cell generation and lipid antigen pulsing

Human CD1 transgene-positive (Tg^+^) and -negative (Tg^−^) bone marrow-derived dendritic cells (BMDCs) were derived from mouse bone marrow progenitors using GM-CSF and IL-4 (PeproTech, Rocky Hill, NJ) as previously described (33). At day 6 of culture, MA was dried out from solvent, resuspended in complete medium and sonicated for 10 min, then BMDCs were harvested and pulsed with free MA or MA-MC at different concentration for 18 h or indicated length of time. MA-pulsed BMDCs were washed twice and used as stimulators to activate DN1 T cells isolated from DN1Tg/hCD1Tg/Rag^−/−^ mice.

### ELISA and cytometric bead array (CBA)

MA-ASMc, V-ASMc, or MA pulsed BMDCs were co-cultured with DN1 T cells for either 24 or 48 h and ELISA or CBA were performed, respectively. For ELISA, 96-well plates were coated overnight with anti-mouse IFN-γ (clone: R4.6A2, Biolegend) at 4 μg/ml, washed and blocked, then incubated with culture supernatant for 2 h followed by detection with biotinylated anti-IFN-γ mAb (clone: XMG1.2) and streptavidin conjugated with alkaline phosphatase (Bio-Rad). The color was developed using substrate pNPP (Sigma). For CBA, GM-CSF, IFN-γ, TNF-α, and IL-17 were measured using CBA Kit (BD Biosciences) according to the manufacturer's instructions. Flow cytometry was performed as described.

### Confocal microscopy

BMDCs were seeded onto poly-L-lysine coated μ-Slide 8 well plates (ibidi) on day 6 of culture. On day 7, cells were pulsed for 4 h with 1 mg/ml of V-ASMc or MA-ASMc. Live cells were treated with 100 nM LysoTracker Red (ThermoFisher Scientific) for 30 min and then imaged on a Leica SP5 II laser scanning confocal microscope.

### *In vivo* imaging

Micelles covalently linked to Dylight 755 were prepared at a polymer concentration of 25 mg/ml and administered either intravenously (i.v.) or intranasally (i.n.). At 3, 24, and 48 h after administration, mice were sacrificed, and various organs were harvested to visualize the biodistribution of micelles by a near-IR *in vivo* Imaging System (IVIS; Center for Advanced Molecular Imaging, Northwestern University) with λ_ex_ = 745 nm, λ_em_ = 810 nm.

### Immunization with MA-loaded PEG-PPS micelles

Mice were immunized i.n. with MA-ASMc containing 1–2 μg of MA in a total volume of 50 μl. Non-immunized or V-ASMc-immunized mice were used as controls. Mice were sacrificed for the detection of MA-specific T cell response at day 6 post-immunization for DN1 T cell-transferred recipients or at day 7 post-immunization for wildtype mice.

### Adoptive transfer and proliferation assay

MA-specific TCR transgenic DN1 T cells were isolated from the spleen and lymph nodes of DN1Tg/hCD1Tg/Rag^−/−^ mice and labeled with CellTrace Violet (ThermoFisher Scientific) as per manufacturer's instructions. 1 × 10^6^ DN1 T cells were adoptively transferred to CD45.1 congenic hCD1Tg mice i.v. 1 day before immunization. Mice were sacrificed 6 days after immunization, and lymphocytes isolated from lungs, spleens, and lymph nodes were used to detect the activation and proliferation of DN1 T cells by flow cytometry.

### IFN-γ ELISPOT assay

IFN-γ ELISPOT assay was performed as previously described (14), with some modifications. Briefly, multiscreen-IP plates (Millipore, Bedford, MA) were coated with anti-IFN-γ mAb (An-18, eBioscience) at 5 μg/ml in PBS. Lymphocytes from immunized mice were incubated with hCD1Tg^+^ or hCD1Tg^−^ BMDCs pre-pulsed with or without MA for 18 h at 37°C. Plates were washed using PBS/0.05% Tween 20 and developed using biotinylated anti-IFN-γ mAb (R4.6A2, eBioscience), followed by streptavidin-conjugated alkaline phosphatase (Jackson ImmunoResearch Laboratories, West Grove, PA) and a BCIP/NBT substrate kit (Bio-Rad, Hercules, CA) according to the manufacturer's instruction. IFN-γ-producing cells were quantified using an ImmunoSpot reader (Cellular Technology, Shaker Heights, OH).

### Statistical analysis

Statistical analyses were performed using Prism software 5.0 (GraphPad, La Jolla, CA). When comparing experimental values from two groups of mice, two-tailed student's *t*-tests were used. When comparing experimental values from multiple groups, one-way ANOVA Bonferroni post-tests were used. Statistically significant differences are noted (^****^P < 0.0001; ^***^P < 0.001; ^**^P < 0.01; ^*^P < 0.05).

## Results

### Generation and characterization of mycolic acid-loaded micelles

**Figure 1:**
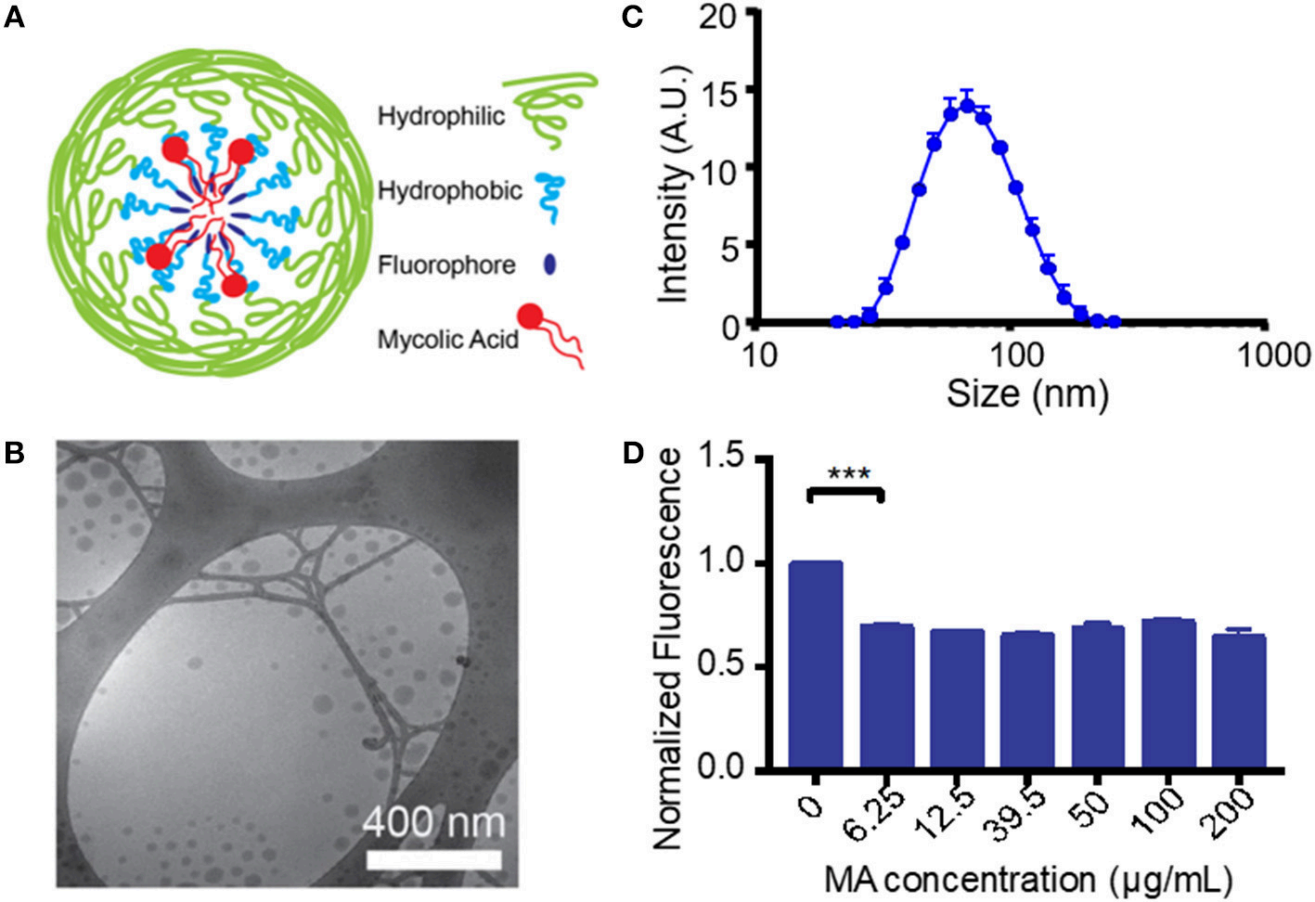
Generation and characterization of mycolic acid-loaded micelles. **(A)** The structure of mycolic acid (MA) loaded PEG-PPS-ASF micelles (MA-ASMc) is represented as a cartoon. Load of MA results in protonation of the fluorophore to decrease fluorescence. **(B)** Representative images of MA-ASMc nanocarriers visualized by cryogenic transmission electron microscopy. **(C)** Dynamic light scattering measurements of MA-ASMc hydrodynamic diameter. Error bars represent standard deviation (SD), *n* = 6. **(D)** Micelles were made with different loadings of MA and fluorescence was measured. Micelles at all concentration of MA > 6.25 μg MA/10 mg PEG-PPS-ASF (1:558 molar ratio) showed a 30% decrease in fluorescence. ***P < 0.001.

Unlike most protein antigens, MA has limited solubility in water, making delivery to APCs particularly difficult. To overcome this challenge and increase the effective dose of MA, we encapsulated MA into a micellar nanocarrier (MA-Mc) using the controlled self-assembly of PEG-PPS, which can form diverse nanocarrier morphologies to efficiently deliver hydrophobic and hydrophilic moieties to APCs (21). To track the intracellular release of MA from micelles following uptake by cells, we modified the PEG-PPS copolymer by attaching an acid sensitive fluorophore (ASF, λ_ex_ = 395 nm, λ_em_ = 505 nm) (29) to the terminal end of the PPS block (PEG-PPS-ASF; Figures S1,S2A). The ASF contains an aminoquinoline ring, and the protonation of the tertiary amine within the ring leads to 98% quenching of fluorescence (Figure S1) (30). We assembled MA-ASMc from this copolymer, where MA was loaded into the core of the nanocarriers (Figure 1A). The spherical morphology of MA-ASMc was confirmed by cryo-transmission electron microscopy (cryoTEM; Figure 1B). The hydrodynamic diameter of MA-ASMc was measured by dynamic light scattering (DLS) to be 68 nm, a size comparable to the unloaded vehicle (V-ASMc), with a zeta potential of −16.5 (Figure 1C,Table 1). The loading of MA led to a 30% decrease in the fluorescence intensity of MA-ASMc compared to V-ASMc (Figure 1D), and this decrease was consistent in solutions with pH values of 4 and above (Figure S2B).

**Table 1:** Properties of self-assembled PEG-PPS-ASF micelles with and without loading of mycolic acid.

	**Size**	**PDI**	**Zeta potential**
Empty Micelle	66.52	0.153	−7.26
MA-loaded Micelle	68.13	0.140	−16.5

As MA does not absorb light at any UV-Visible wavelength, to determine the loading efficiency of MA in PEG-PPS micelles, MA was first conjugated to 4-bromomethyl-6,7-dimethoxycoumarin and then loaded into PEG-PPS nanocarriers. After purification, the fluorescence of the coumarin derivative was measured. The loading efficiency of MA into micelles was 92 ± 3% on average when 100 μg of coumarin-conjugated MA was used as a payload within 10 mg of PEG_44_-PPS_15_-ASF micelles (Figure S3). This is significantly higher than the previously-reported 2% loading efficiency for poly(lactic-co-glycolic acid; PLGA) nanocarriers (34). The predicted partition coefficient (logP) for alpha-MA (the most common mycolic acid) is 10.66, a value that is greater than the 9.056 logP of indocyanine green, which we previously found to have a 97% loading efficiency in PEG-PPS nanocarriers (18). Such a high loading efficiency is expected for a molecule with high solubility in non-polar solvents, and demonstrates the ability of these micelles to package a highly hydrophobic antigen for *in vivo* delivery. Unless otherwise stated, this ~1:35 molar ratio of MA to PEG-PPS-ASF was used for all subsequent experiments for consistency.

### MA-Mc are endocytosed by BMDCs and display superior efficacy over free MA in activating CD1b-restricted MA-specific TCR transgenic (DN1) T cells

**Figure 2:**
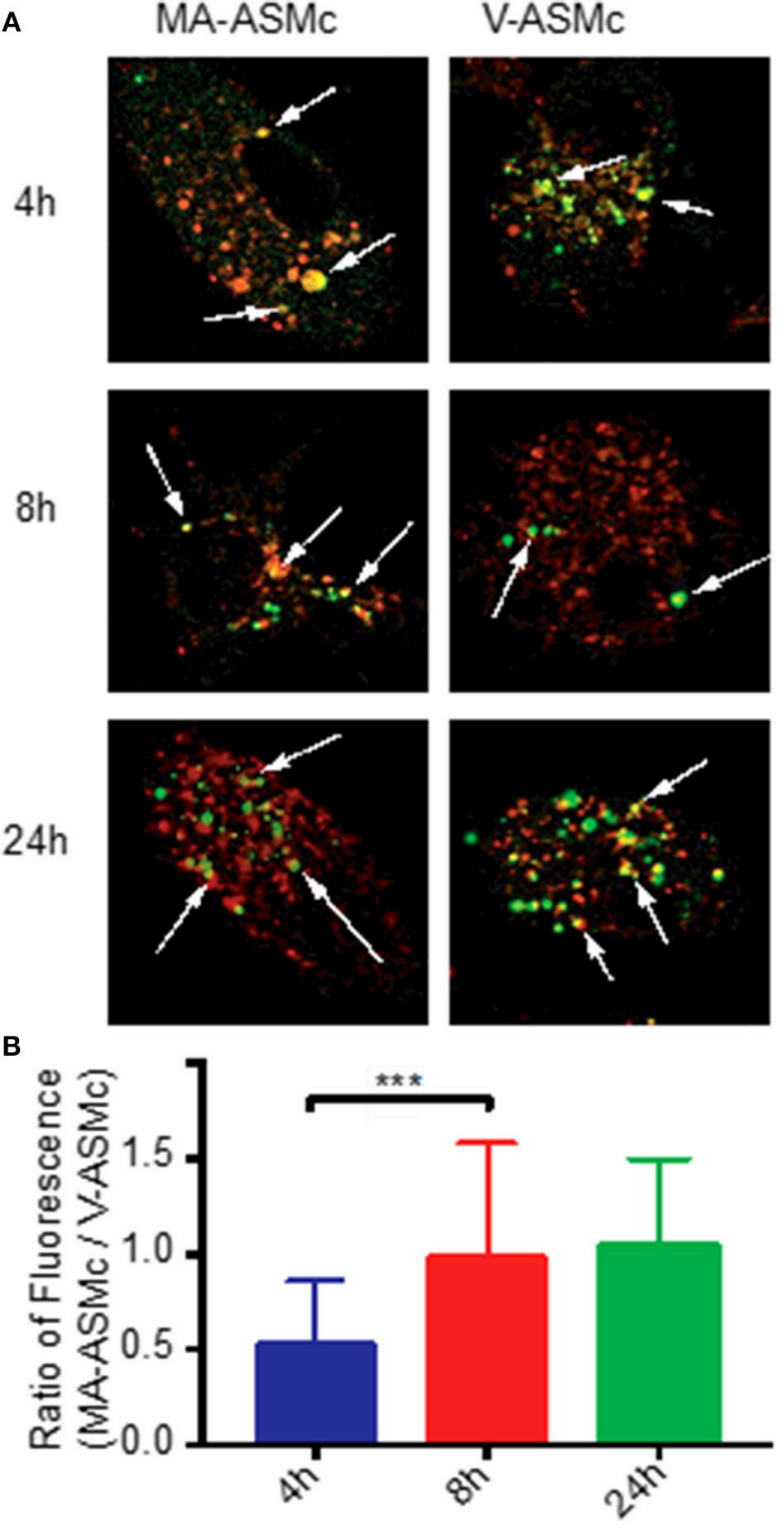
PEG-PPS-ASF functions as an on/off fluorescent switch to indicate intracellular release of MA following endocytosis by BMDCs. **(A)** Live BMDCs were imaged by confocal microscopy at different time points after pulsing with 1 mg/ml of either MA-ASMc or vehicle micelles (V-ASMc) for 4 h. Lysosomes were stained with Lysotracker (red); Micelles appear in green. Magnification is 100 × . Arrows indicate points of co-localization (yellow). **(B)** The fluorescence intensity of cells incubated with either MA-ASMc or V-ASMc was measured at time points of 4, 8, and 24h. The ratio of cell fluorescence (MA-ASMc / V-ASMc) significantly increased and remained stable after 4h, suggesting release of MA from nanocarriers between 4 and 8 h. ***P < 0.001.

For CD1-restricted T cell activation, MA-Mc must be internalized by CD1b-expressing BMDCs followed by release of MA from the nanocarriers into their lysosomal compartments. To track both the release of MA from nanocarriers and the uptake of the PEG-PPS copolymer, we live imaged BMDCs at different timepoints after pulsing with V-ASMCs and MA-ASMc. At all time-points assayed, co-localization was observed between the lysosome and the nanocarriers (Figure 2A).

To measure differences in intracellular fluorescence between V-ASMc and MA-ASMc, we normalized the fluorescence intensity of the nanocarriers to the background cytosol fluorescence of each cell, excluding the endosomal punctate. After 4 h, there was a significantly higher fluorescence intensity observed for cells incubated with V-ASMc compared to those incubated with MA-ASMc at the same cell and micelle concentrations, resulting in a low MA-ASMc/V-ASMc cell fluorescence ratio (Figure 2B). The MA-ASMc/V-ASMc cell fluorescence ratio significantly increased after 8 h but no significant change was observed for the 24 h timepoint, indicating that MA was likely released from the nanocarriers between 4 and 8 h after uptake.

**Figure 3:**
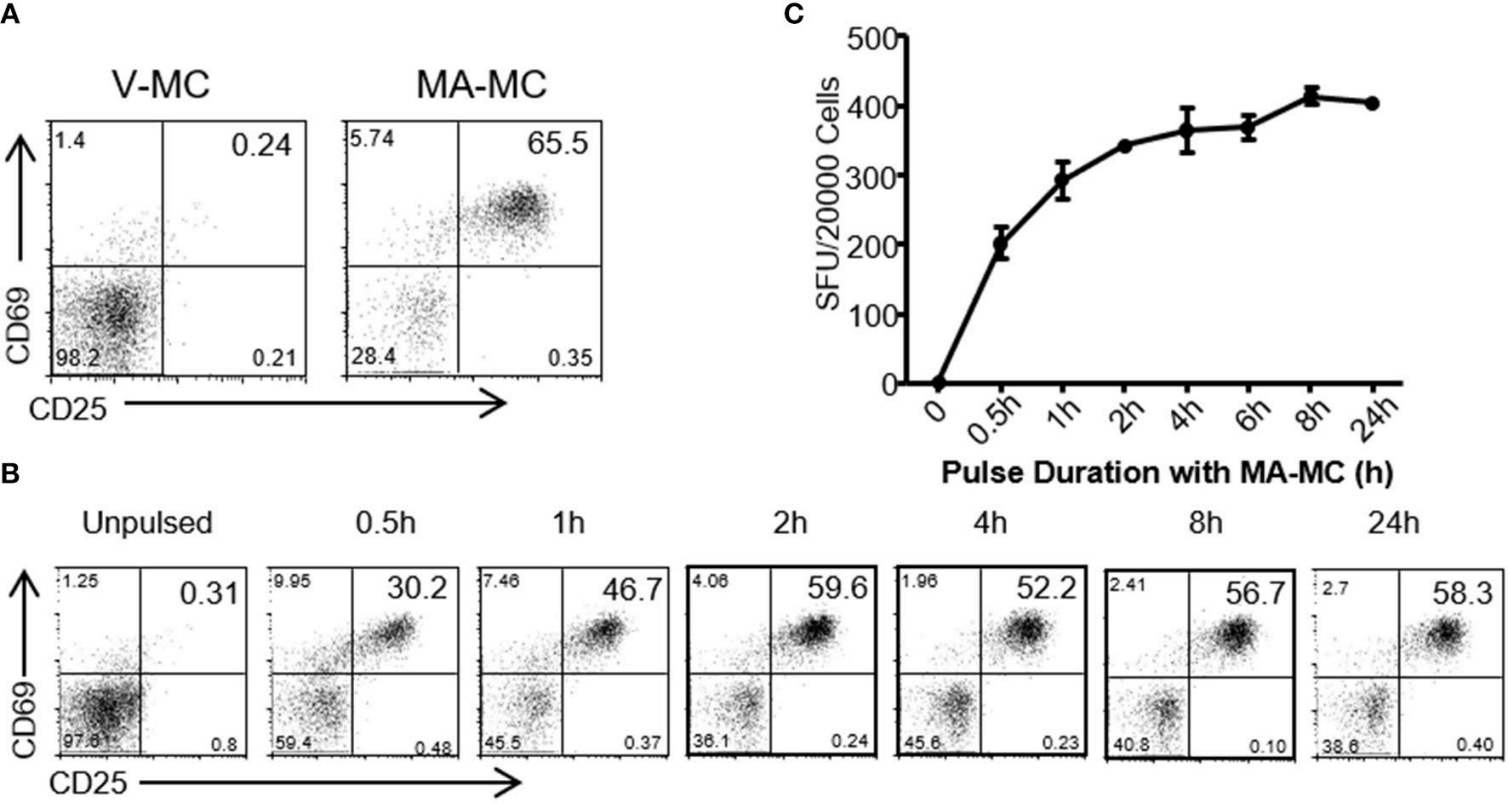
MA-ASMc are quickly endocytosed by BMDCs to activate MA-specific TCR transgenic T cells. Bone marrow derived dendritic cells (BMDCs) from hCD1Tg mice were pulsed with MA-ASMc at 0.2 μg/ml for different length of times and then co-cultured with MA-specific TCR transgenic T cells (DN1) for 24 h to determine the length of time needed to efficiently activate DN1 T cells by flow cytometry and IFN-γ ELISPOT. **(A)** Representative dot-plot of activated DN1 cells expressing CD69 and CD25 responding to MA-ASMc stimulation. **(B)** The percentage of CD25^+^CD69^+^ DN1 T cells activated by BMDCs pulsed with MA-ASMc for different length of times. **(C)** The number of IFN-γ-producing DN1 T cells activated by BMDCs pulsed with MA-ASMc for different length of time.

To assess whether MA released from MA-ASMc was processed and presented by CD1b-expressing APCs, we examined whether hCD1Tg^+^ BMDCs could activate MA-specific T cells after uptake of MA-ASMc (Figure 3). hCD1Tg^+^ BMDCs were pulsed with MA-ASMc for different lengths of time, their ability to activate CD1b-restricted MA-specific TCR transgenic DN1 T cells was measured by flow cytometry and ELISPOT assay to respectively, quantify T cell expression of activation markers CD69 and CD25 as well as secretion of IFN-γ (Figures 3B,C). While V-ASMC pulsed hCD1Tg^+^ BMDCs did not activate DN1 T cells (Figure 3A), MA-ASMc-pulsed hCD1Tg^+^ BMDCs activated DN1 T cells to their maximal level within 4 h of pulsing (Figures 3B,C), which correlates well with the timing of intracellular MA release indicated by the cell fluorescence measurements (Figure 2B). In addition, pulsing with V-ASMC or MA-ASMc did not upregulate the expression of CD1b and DC maturation markers (CD80 and CD86; Figure S4), indicating non-immunomodulatory nature of PEG-PPS copolymer. Overall, these results verified that MA-ASMc effectively delivered MA to CD1b-expressing hCD1Tg^+^ BMCDs for Ag presentation.

**Figure 4:**
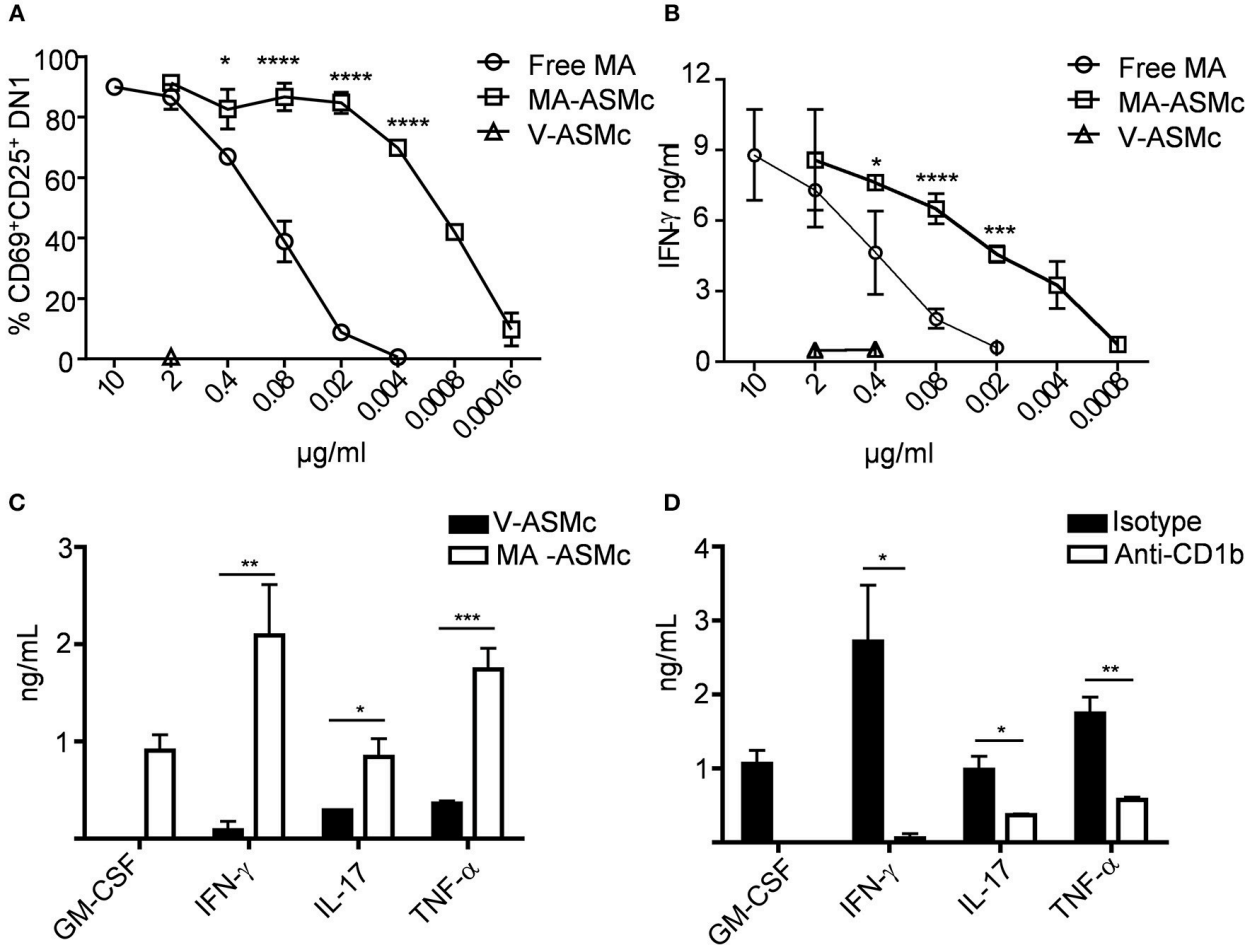
MA-ASMc are superior to free MA in activating MA-specific TCR transgenic T cells and elicit cytokine expression. BMDCs from hCD1Tg mice were pulsed with serial dilutions of MA-ASMc, free MA and V-ASMc overnight and then co-cultured with MA-specific TCR transgenic T cells (DN1) for 24 h. The efficiency of MA-ASMc and free MA in activating DN1 T cells was compared by flow cytometry and IFN-γ ELISA. **(A)** The percentage of CD25^+^CD69^+^DN1 T cells activated by different concentrations of MA-ASMc, free MA and V-ASMc; (*n* = 4); **(B)** The concentration of IFN-γ produced by DN1 T cells in response to stimulation of different concentrations of MA-ASMc, free MA and V-ASMc; (*n* = 4). **(C,D)** CBA cytokine analysis of DN1 stimulated for 48 h with hCD1Tg MHC II deficient BMDC pulsed with **(C)** MA-ASMc or V-ASMc or **(D)** pulsed with MA-ASMc and treated with CD1b Ab or isotype control; (*n* = 4). *P < 0.05; **P < 0.01; ***P < 0.001; ****P < 0.0001.

To compare the efficacy of MA-ASMc and free MA in activating MA-specific T cells, hCD1Tg^+^ BMDCs were pulsed with various concentrations of free MA and MA-ASMc prior to co-culture with MA-specific DN1 T cells. Following 24 h of co-culture, the expression of activation markers (CD69 and CD25) and production of IFN-γ by DN1 T cells were determined by flow cytometry and ELISA, respectively. We found that while DCs pulsed with free MA were able to activate DN1 T cells as reflected by upregulation of activation markers (Figure 4A) and IFN-γ production (Figure 4B), an ~100 fold lower and ~20 fold lower respective concentration of MA was required to activate DN1 T cells to similar level when MA was delivered in the form of MA-ASMc (0.02 μg/ml MA in MC vs. free MA). As a negative control, DCs pulsed with V-ASMc did not activate DN1 T cells (Figures 4A,B), further confirming the non-immunogenic nature of PEG-PPS copolymer. We characterized additional cytokines produced by DN1 T cells using CBA in the supernatant of a 48 h co-culture of DN1 T cells with either V-ASMc or MA-ASMc-pulsed hCD1Tg^+^ BMDCs. We found DN1 T cells secreted GM-CSF, IFN-γ, TNF-α, and IL-17 in response to stimulation with MA-ASMc-pulsed hCD1Tg^+^ BMDCs (Figure 4C). Cytokine production was CD1b dependent, as the response was blocked by an anti-CD1b antibody (Figure 4D). These data indicated that encapsulation within PEG-PPS micelles greatly enhances the antigen presentation of MA by DCs. Furthermore, MA-ASMc elicits CD1b-dependent production of GM-CSF, IFN-γ, TNF-α, and IL-17 (Figure 4D).

### MA-Mc are retained in the lung and taken up by alveolar macrophages and myeloid DCs after intranasal immunization

**Figure 5:**
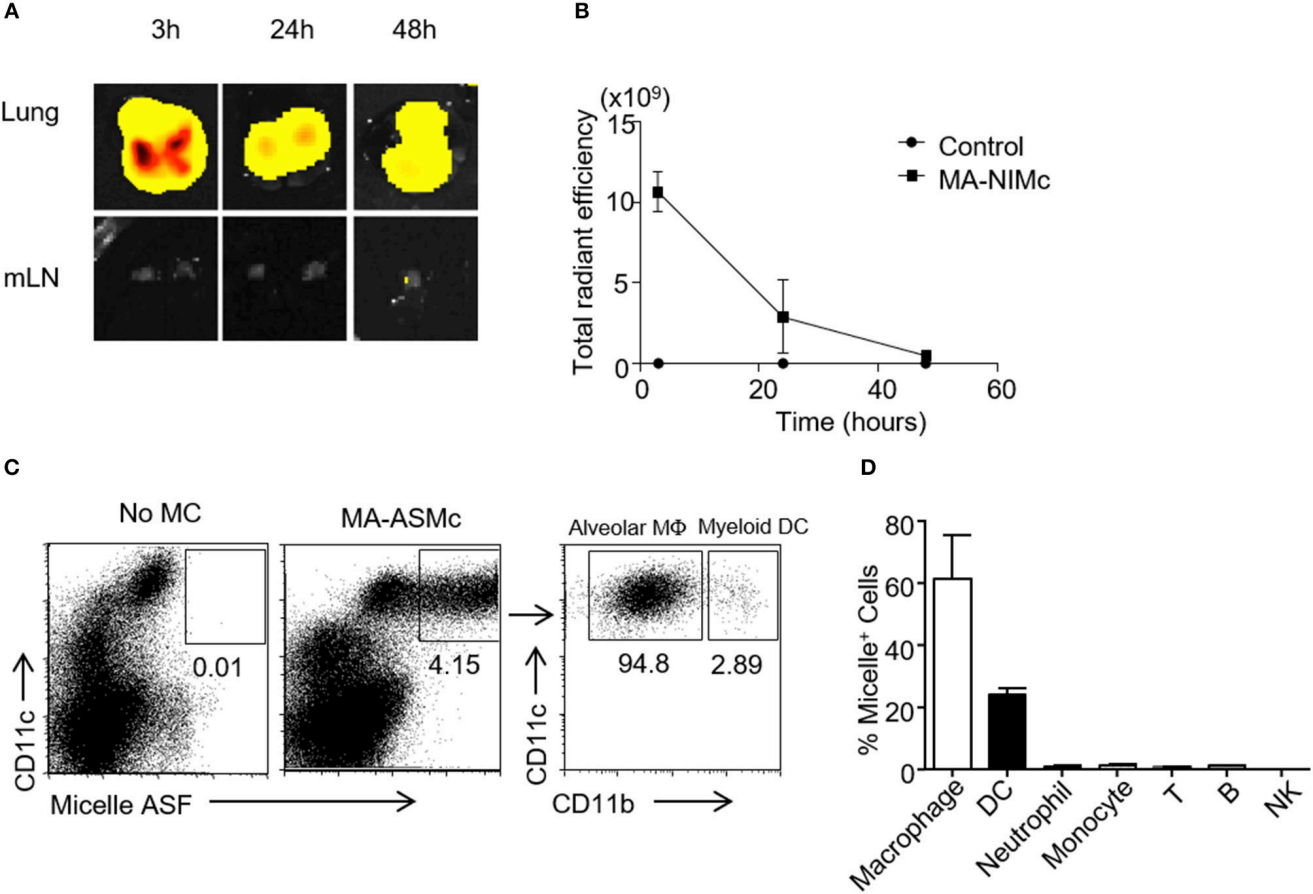
MA-Mc are mainly retained in the lung after pulmonary delivery and taken up by alveolar macrophages and myeloid DCs. The *in vivo* bio-distribution of Dylight 750 conjugated (NIMc) or ASF-labeled micelles (ASMc) in different organs was visualized by *In Vivo* imaging system (IVIS) **(A,B)** and tracked by flow cytometry **(C,D)** after pulmonary administration. **(A,B)** The kinetics **(A)** and intensity **(B)** of MA-NIMc in the lung and mediastinal lymph nodes (mLN) were visualized by IVIS from 3 to 48 h after i.n. delivery. **(C)** MA-ASMc-carrying cells in the lungs of unimmunized vs. immunized mice were examined by flow cytometry. **(D)** The percentage of different leukocyte subsets carrying MA-ASMc in the lung of mice (*n* = 3) 24 h after pulmonary delivery. Data are representative from three repeat experiments.

In past studies utilizing BCG, optimal protection has been achieved when the BCG vaccine is administered directly to the pulmonary mucosa, and it is generally established that the route of vaccine administration should follow the route of infection (35, 36). Therefore, we studied the induction of MA-specific T cell responses in the lung following pulmonary delivery of MA-MCs *via* the intranasal (i.n.) route. Our previous studies showed that PEG-PPS micelles could be taken up non-specifically by cells of the mononuclear phagocytes system (MPS) and quickly removed from circulation after intravenous injection (23). The biodistribution of MA-loaded micelles following i.n. delivery had not yet been determined, and it was not known whether they would also be rapidly removed from circulation following i.n. administration. To address these questions, PEG_44_*-*PPS_15_ micelles were covalently linked to Dylight 755 *via* a thiol-maleimide conjugation for whole organ IVIS imaging. After i.n. administration, the biodistribution of micelles in different organs was assessed by NIRF imaging. We found that empty/vehicle Dylight 755-labeled micelles (V-NIMC) were mainly retained in the lung and reduced gradually from 3 to 48 h post administration (Figures 5A,B). The signal in the mediastinal lymph nodes (MLN), axillary lymph nodes (AxLN), spleen, liver and kidneys was barely detectable (Figure S5). Therefore, although we have previously demonstrated that i.v. injection of PEG-PPS micelles targets multiple organs (23), the i.n. route was found to only target the lung at time points of 3, 24, and 48 h after administration.

To further investigate which subset of cells are responsible for the uptake of these micelles, we also intranasally administered the MA-ASMc. As described above, the fluorescence of the ASF conjugated to micelles can be readily detected within cells by flow cytometry after intracellular delivery. Single cell suspensions were prepared from the lung, MLN and spleen at 3 and 12 h after i.n. delivery of MA-ASMc and cells positive for micelle fluorescence were examined by flow cytometry. We found that MA-ASMc were taken up selectively by a population of CD11c^+^ cells in the lung (Figure 5C), which included mostly alveolar macrophages (SiglecF^+^CD11c^+^CD11b^−^) and a small percentage of myeloid DCs/interstitial macrophages (CD11b^+^CD11c^+^; Figure 5C) whereas T cells, B cells, NK cells, neutrophils and monocytes did not contain MA-ASMc (Figure 5D). Consistent with the results from IVIS, no MA-ASMc containing cells could be detected in mLN and spleen (Figure S6). These results suggested that MA-MCs were selectively phagocytosed by APCs like alveolar macrophages and myeloid DCs in the lung after intranasal delivery.

### Intranasal delivery of MA-Mc induces proliferation and activation of adoptively-transferred MA-Specific T cells

**Figure 6:**
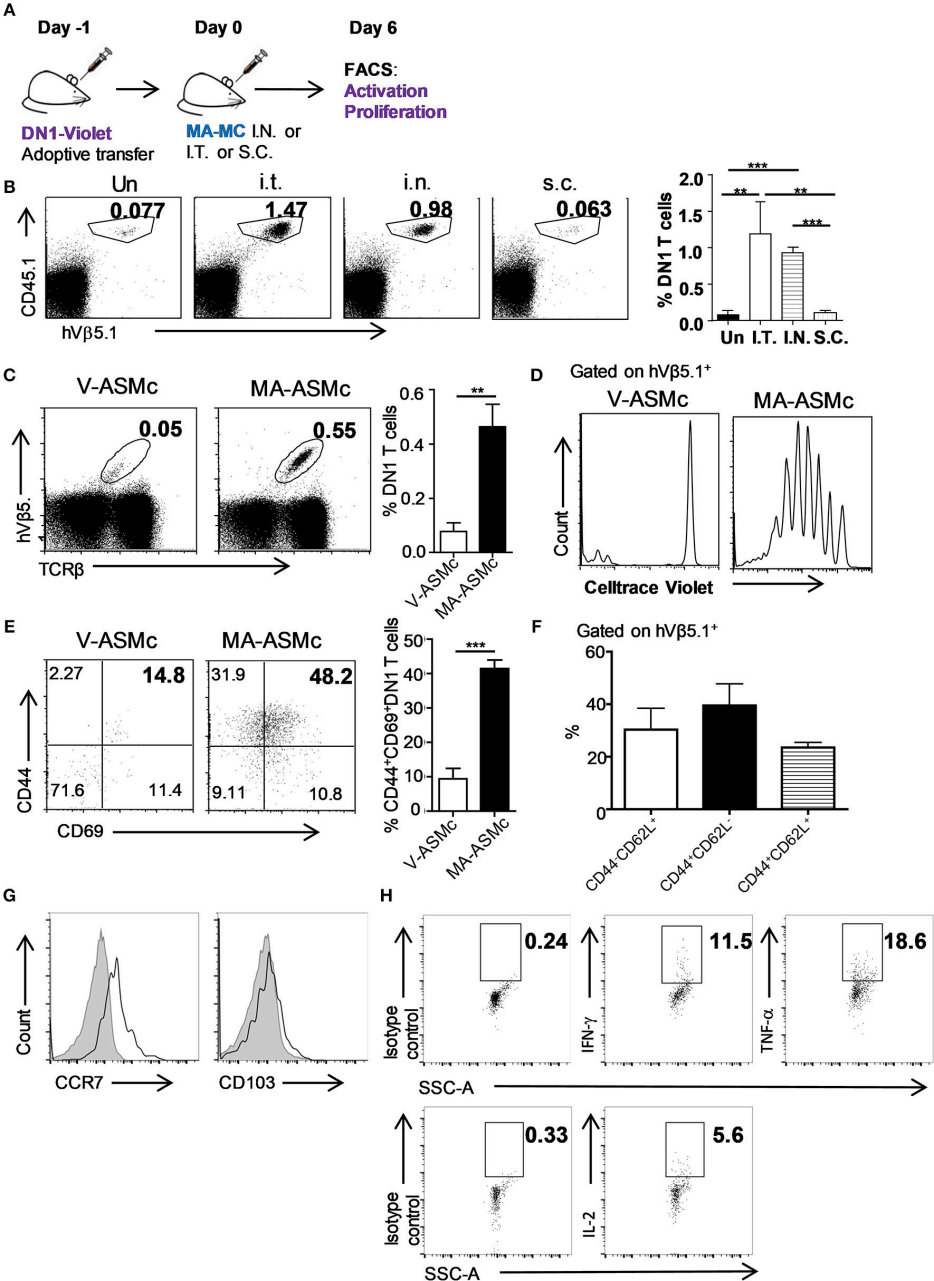
i.n. delivery of MA-ASMc induces proliferation and activation of adoptively-transferred MA-specific T cells. MA-specific T cells (DN1) were labeled with Celltrace violet and adoptively transferred into hCD1Tg mice 1 day before immunization with MA-ASMc or micelle vehicle (V-ASMc) via different routes. Six days later, DN1 T cells were harvested from V-ASMc-, or MA-ASMc-immunized or unimmunized hCD1Tg mice for detection of proliferation and activation. **(A)** Schematic diagram of experimental design. **(B)** Representative dot plots and percentage of DN1 T cells in the MLN of recipient mice unimmunized via intratracheal (I.T., *n* = 4), intranasal (I.N., *n* = 3) and subcutaneous (S.C., *n* = 4) route. **(C)** Representative dot plot of DN1 T cells from MLNs of V-ASMc (*n* = 5) vs. MA-ASMc-immunized (*n* = 6) hCD1Tg mice. **(D)** Proliferation and **(E)** activation of DN1 T cells were compared in V-ASMc (*n* = 3) vs. MA-ASMc-immunized (*n* = 4) hCD1Tg mice by flow cytometry. Data are representative of three experiments. **(F)** Percent expression of CD44/CD62L and **(G)** representative histogram of CCR7 and CD103 expression (black line) on DN1 T cells in the lung (*n* = 4). Gray solid areas indicate isotype controls. **(H)** Representative intracellular cytokine staining of IFN-γ, TNF-α, and IL-2 in DN1 T cells from the spleen after phorbol 12-myristate 13-acetate (PMA) and ionomycin (INO) stimulation (*n* = 4). *P < 0.05; **P < 0.01; ***P < 0.001.

After determining that MA-loaded PEG-PPS micelles can be efficiently delivered to APCs in the lung following intranasal immunization, we next performed *in vivo* antigen presentation assays to determine whether MA-ASMc phagocytosed by APCs can activate MA-specific DN1 T cells *in vivo* (Figure 6A). We also tested whether other routes of administration could be a better option than the intranasal route. CellTrace violet-labeled DN1 T cells were adoptively transferred into hCD1Tg mice. One day later, recipient mice were immunized by intranasl (i.n.), intratracheal (i.t.), and subcutaneous (s.c.) route with MA-ASMc. At day 6 post-immunization, we determined the proliferative capacity and activation status of DN1 T cells in the lung, MLN and spleen by flow cytometry. Compared to s.c. or no immunization, i.n. and i.t. induced significantly higher percentages of DN1 T cells recovered from MLN, while there was no significant difference in DN1 T cell level between i.n and i.t. immunized groups (Figure 6B). Considering the invasiveness and higher technical requirement of i.t., our data supports i.n. delivery to be the most practical option for delivery of MA-ASMc. After i.n. immunization, MA-ASMc-immunized mice had a higher percentage of DN1 T cells than V-ASMc-immunized mice in MLN (Figure 6C), lung and spleen (Figure S7), although MA-ASMc-carrying DCs were not readily detectable in lymph node and spleen by flow cytometry (Figure S6). In addition, DN1 T cells in MA-ASMc-immunized mice underwent extensive proliferation (Figure 6D) and were significantly more activated, with a higher percentage of cells expressing CD44^hi^CD69^+^, compared to those in V-ASMc-immunized mice (Figure 6E). DN1 T cells in MA-ASMc-immunized mice displayed an effector phenotype, as the majority are CD44^hi^CD62L^−^ with some CCR7 expression, but no CD103 expression in the lung (Figures 6F,G). PMA/ionomycin-stimulated DN1 T cells were also able to produce IFN-γ, TNF-α, and IL-2 (Figure 6H). These results demonstrate that pulmonary delivery of MA-ASMc leads to the presentation of MA by APCs and subsequent activation of MA-specific T cells *in vivo*.

### Intranasal immunization of MA-Mc elicits polyclonal MA-Specific T cell responses in hCD1Tg^+^ Mice

**Figure 7:**
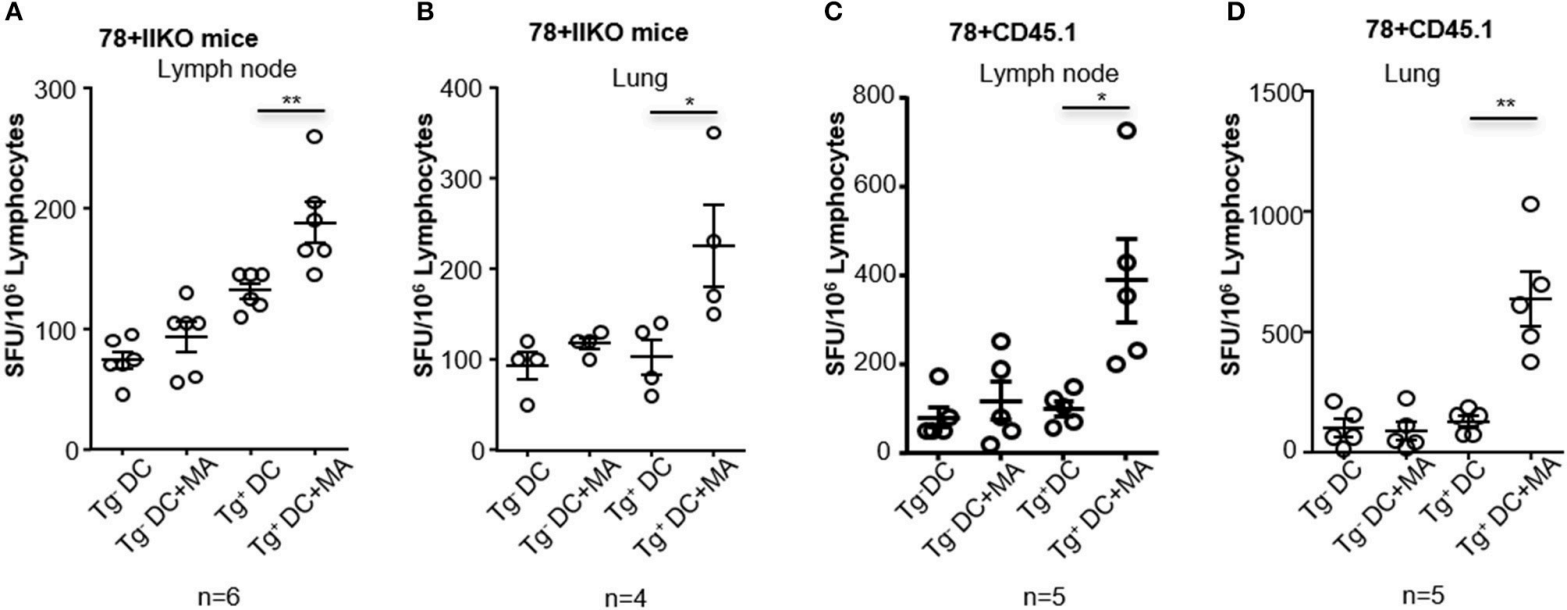
Intranasal immunization with MA-ASMc induces MA-specific T cell response in hCD1Tg mice. hCD1Tg mice in MHC class II-deficient (**A,B**; *n* = 4–6) or wildtype background (**C,D**; *n* = 5) were immunized intranasally with MA-ASMc at a dosage of 1 μg mycolic acid. Mice were sacrificed 1 week later and lung **(B,D)** and lymph node **(A,C)** were harvested to make single cell suspension. Human CD1 transgenic and mycolic acid-specific T cell responses were detected in lung and lymph node in response to re-stimulation with mycolic acid pulsed or un-pulsed hCD1Tg negative (Tg^−^) or positive (Tg^+^) BMDCs in an IFN-γ ELISPOT assay. *P < 0.05; **P < 0.01.

Although adoptively transferred MA-restricted DN1 T cells could be activated *in vivo* after immunization with MA-ASMc, there are more physiologically relevant ways to probe MA-loaded micelle immunogenicity. MA-specific T cells in hCD1Tg mice are polyclonal, have a more diverse TCR repertoire and are less frequent than adoptively transferred DN1 T cells. Therefore, to determine if MA-ASMc immunization could induce polyclonal MA-specific T cell responses in hCD1Tg mice, we immunized hCD1Tg mice i.n. with MA-ASMc and detected MA-specific responses by an IFN-γ ELISPOT assay. hCD1Tg^+^ mice in both wildtype (B6) and MHC II-deficient (II^−/−^) background were used for immunization, as our previous study showed that hCD1Tg/II^−/−^ mice exhibited a more consistent group 1 CD1-restricted T response upon immunization with MA-pulsed DCs (14). At day 7 post-immunization, lymphocytes were isolated from the lung and MLN of MA-MC-immunized mice and stimulated *in vitro* with unpulsed or MA-pulsed BMDCs from hCD1Tg/II^−/−^ (Tg^+^) or II^−/−^ (Tg^−^) mice. Compared to stimulation with MA-pulsed Tg^−^ DCs or unpulsed DCs, ELISPOT assays revealed that lymphocytes from both strains of immunized hCD1Tg^+^ mice had a significantly higher number of IFN-γ-producing cells when stimulated with MA-pulsed Tg^+^ DCs (Figure 7). This data indicates that pulmonary delivery of MA-loaded PEG-PPS micelles efficiently elicit group 1 CD1-restricted MA-specific T cell responses in the lung and MLN of both hCD1Tg^+^ and hCD1Tg/II^−/−^ mice.

## Discussion

As CD1 molecules present many lipid antigens derived from Mtb and are non-polymorphic, CD1-restricted Mtb lipid antigens are likely to be recognized by most individuals, making them attractive vaccine targets and an untapped mechanism of improving immunity (15). In this study, using MA, a major component of the Mtb cell wall, we developed a MA-loaded micellar nanocarrier amenable to pulmonary administration and capable of significantly enhancing CD1b-restricted T cell responses both *in vitro* and *in vivo*. Our use of polymeric micelles self-assembled from ASF-conjugated PEG_44_*-*PPS_15_ provided evidence that this enhanced activation may be due to more efficient delivery of MA to APC endosomes for antigenic processing. Using human group 1 CD1-expressing mice generated in our lab, we demonstrated that MA-loaded PEG-PPS micelles can be taken up by APCs *in vitro* and *in vivo*, and subsequently elicit MA-specific CD1b-restricted T cell response in hCD1Tg^+^ mice in both monoclonal and polyclonal settings after pulmonary delivery. Our results support the notion that Mtb lipid antigen can be harnessed to develop vaccines by targeting group 1 CD1-resticted T cells.

Although a ~35:1 molar ratio of PEG_44_-PPS_15_-ASF: MA was consistently obtained, the maximum decrease in fluorescence of the ASF in the presence of loaded MA occurred at a higher molar ratio (e.g., 558:1), likely due to MA being a weak acid and the fluorophore being hindered within the micelle core. This, along with an insensitivity to pH ranges within lysosomes (pH 4.5-5.5), made PEG_44_-PPS_15_-ASF advantageous for detection of MA release within cells following endosomal delivery. As previously employed in non-biological systems (30), the naphthalimide-based dye thus functioned as an on/off fluorescent signal, here indicating when ~93% or more of MA had been released from micelles. To assess organ-level biodistributions of MA-Mc following intranasal administration, the ASF was replaced with a lipophilic NIRF-sensitive fluorophore. Conjugation of lipophilic fluorophores to the hydrophobic PPS terminal end of PEG-PPS copolymers sequesters the fluorophore, as well as most non-conjugated lipophilic payloads, within the micelle core upon self-assembly (18, 23). Thus switching the hydrophobic tag does not impact the nanocarrier biodistribution, which is instead dictated by the chemistry of the outer PEG corona and nanostructure morphology (23).

The most abundant lipid component of Mtb is MA, however human group 1 CD1 molecules present a large array of Mtb-derived lipids to cognate T cells. Although the relative immunodominance of lipid antigen during Mtb infection is currently unknown, a few Mtb lipid-based liposome vaccines have been explored either *in vitro* or *in vivo*. A glucose monomycolate (GMM)-loaded liposome vaccine was delivered to human DCs, which induced robust activation of GMM-specific human T cell clones *in vitro* (37). Lipoarabinomannan (LAM)-loaded liposomes triggered LAM-specific human T lymphocytes response *in vitro* (38). However, these studies were not further tested in animal models that express group 1 CD1 molecules. A mycobacterial total lipid-based liposome has been tested in guinea pigs that express conserved group 1 CD1 isoforms. Although total lipid-based liposomes induced CD1 restricted T-cell responses and demonstrated an improved pulmonary pathology (39, 40), the specific lipid component among the total lipids that was responsible for the pathological protection was not clear. Recently, a diacylated sulfoglycolipids (Ac_2_SGL) and phosphatidylinositol mannoside 2 (PIM_2_)-loaded liposome vaccine induced protective immune responses in guinea pigs (41), but the adjuvant effect of PIM_2_ and trehalose-6,6-dibehenate (TDB), a component of the liposomal nanocarrier (42, 43), may complicate the bacterial and pathological protection observed.

The need to stably package lipids for increased delivery efficiency arises from their inherent hydrophobicity. As described above, liposomes have been employed as a delivery vehicle for several Mtb lipid-based vaccines (37–39, 44), but polymer-based nanocarriers provide advantages of increased stability (45), ease of preparation (18, 46), and control over bioresponsive payload delivery and targeting (19–23, 47). In our previous studies, self-assembled solid core nanocarriers, and polymersomes assembled from PEG-PPS copolymers were shown to be an effective intracellular delivery system for protein antigens and adjuvants to enhance the induction of T cell immunity (20, 21). Here, our results demonstrated that PEG–PPS micelles are also an efficient delivery system for lipid antigens, achieving high loading efficiency, intracellular delivery of lipid antigen into the lysosome for CD1 receptor complexation, and amenability to i.n. administration for elicitation of localized CD1-restricted T cell responses. We selected MA as the lipid antigen because it stimulates potent cytokine production from CD1b-restricted human T cells (2), which contributes to the acute response of Mtb infection in humans and induces memory responses upon *ex vivo* re-stimulation in drug-treated TB patients (10). Importantly, MA-specific DN1 T cells were demonstrated to confer protection against Mtb infection in hCD1Tg mice (13). We hypothesized that combining (1) the efficient intracellular delivery of MA *via* our stable PEG-PPS nanocarrier platform and (2) *in vivo* evaluation of elicited CD1-restricted T cell responses in our unique hCD1Tg mice would provide a means for the rational design and optimization of subunit vaccines incorporating lipid antigens.

Although Mtb lipid-based liposomes have been tested in guinea pigs (39, 40, 44), *in vivo* biodistribution information was lacking. We therefore synthesized and assembled MA-NIMc to evaluate organ level biodistributions following i.n administration. MA-NIMc were not visible in organs outside the lung from 3 to 48 h after pulmonary delivery as assessed by IVIS imaging (Figure 4). Flow cytometric analysis following i.n. administration of MA-ASMc revealed uptake mostly by alveolar macrophages but not by non-phagocytic cells. As CD1b is only expressed on DCs in the periphery (14, 15), the activation of MA-specific T cells detected *in vivo* in this study is unlikely due to direct presentation of MA/CD1b by macrophages, and may instead occur through cross presentation of MA by CD1b-expressing DCs that phagocytosed apoptotic macrophages (48). An earlier study showed that apoptotic vesicles containing Mtb antigens from mycobacteria infected-macrophages can be taken up by DCs, which present these antigens to T cells through MHC-I and CD1 molecules (49). Consistent with this report, we previously found that DN1 T cells are activated by Mtb-infected DCs but not by Mtb-infected macrophages (13). In the present study, DN1 T cells proliferated best in the MLN though MA-ASMc were not detected in organs outside the lung, suggesting that antigen transfer from MA-ASMc-carrying alveolar macrophage to migratory DCs occurred *in vivo*.

Of note, an MA-CD1b tetramer was developed recently for detecting MA-specific T cell responses in humans (50), however, it has not been validated for use in Mtb-infected animals. Therefore, we primarily detected MA-specific T cell responses by IFN-γ ELISPOT assay in immunized mice. It has been challenging to detect Mtb lipid-specific T cell responses in hCD1Tg mice after immunization with lipid-pulsed group 1 CD1-expressing BMDCs (14), possibly due to the low precursor frequency and/or inefficient expansion of group 1 CD1-restricted T cells in hCD1Tg mice. Therefore, the successful induction and detection of MA-specific T cells responses in hCD1Tg mice after immunization with MA-MCs is a significant advance toward developing an effective lipid-based vaccine against TB.

We observed that eliciting a T cell response did not require adjuvant. However, our nanocarrier platform lends itself to delivery of many different immunostimulants. Adjuvants for enhancement of responses against lipid-antigen have yet to be identified, and our MA-MC/hCD1Tg system presented here provides an excellent means for extensive screening of adjuvant candidates, as PEG-PPS nanocarriers can be engineered to simultaneously deliver combinations of both hydrophobic and hydrophilic payloads (18, 23, 28). We did not observe any adjuvant effect from the unloaded PEG-PPS V-ASMc or V-NIMc on DC maturation *in vitro* or *in vivo*, which supports our previous findings that PEG-PPS nanocarriers are non-immunogenic and are not sufficient to stimulate the immune system without adjuvanting payloads (18, 19, 21, 23). Future work can include adding synergistic combinations of adjuvants, and testing the delivery of MA with different morphologies of PEG-PPS nanocarriers to distinct APC populations.

In summary, we have demonstrated that MA-MCs can elicit MA-specific T cell responses when delivered i.n. to human group 1 CD1 transgenic mice by packaging MA into a non-immunogenic micellar nanocarrier for enhanced intracellular delivery. The humanized CD1Tg mice employed here will support future experiments to evaluate the protective efficacy of MA-Mc-based subunit vaccines in Mtb challenged mice. Incorporation of lipid-antigens may enhance the efficacy of a wide range of subunit vaccine formulations and provide solutions to challenges facing current immunization strategies. For example, HIV-infected patients mostly suffer from co-infection with Mtb due to depletion of CD4^+^ T cells (1), whereas group 1 CD1-restricted T cells are not affected by HIV infection (10, 51). In fact, in a pilot experiment, we were able to detect MA-specific hCD1-restricted T cell responses in hCD1Tg^+^/CD4^−/−^ mice immunized with MA-ASMc (Figure S8). Thus, targeting group 1 CD1-restricted T cells by vaccination with MA-MC-supplemented vaccines could be particularly beneficial for HIV-infected individuals. Additionally, the inclusion of an immunostimulant in our MA-Mc subunit vaccines remains unexplored and may find utility in boosters to enhance immune responses elicited by established vaccines, such as Bacillus Calmette–Guerin (BCG).

## Author contributions

SS, C-RW, and ES conceived and designed the experiments. SS, DK, LC, EM, DV, YH, and QX performed the experiments. SS, DK, and C-RW analyzed the data. DV, C-RW, and ES contributed reagents, materials, analysis tools. SS, DK, EM, C-RW, and ES wrote the paper.

### Conflict of interest statement

The authors declare that the research was conducted in the absence of any commercial or financial relationships that could be construed as a potential conflict of interest.

## Supplementary material

Figure S1 The Synthesis scheme of a poly(ethylene glycol)-*bl*-poly(propylene sulfide) amphiphilic copolymer tagged with a naphthalimide-derived acid sensitive fluorophore (PEG-PPS-ASF).

Figure S2 Characterization of novel acid sensitive fluorophore. **(A)** The fluorophore has an absorbance maximum at 395 nm and an emission maximum at 505 nm. **(B)** Fluorescence of empty (V-ASMc) or MA-loaded acid sensitive fluorophore tagged micelles (MA-ASMc) was measured at different pH values. Fluorescence drops after pH 3. All error bars represent SD, *n* = 3.

Figure S3 Loading efficiency of mycolic acid. Mycolic acid was conjugated to 4-bromomethyl-6,7-dimethoxycoumarin and then loaded into micelles. Fluorescence of the coumarin was measured after purification on an LH20 size exclusion column. The average loading efficiency of mycolic acid was 92 ± 3 %. Green bar represents range of loading efficiency for samples. Error bars represent SD, *n* = 3.

Figure S4 Expression changes of CD1b, CD80, and CD86 on BMDCs pulsed vs. unplused with empty micelles, MA-ASMc, and Free MA. BMDCs from hCD1Tg^+^ mice were differentiated in the presence of GM-CSF and IL-4 for 6 days and then pulsed or unplused with different concentration of empty micelles, MA-ASMc and Free MA for 24 h. The expression of CD1b, CD80, and CD86 on BMDCs were examined by flow cytometry. Data are expressed as fold changes of mean fluorescence intensity (MFI) of each marker on pulsed (*n* = 3) vs. unpulsed (*n* = 2) BMDCs. Data are pooled from two experiments and expressed as mean.

Figure S5 MA-NIMc are mainly retained in the lung after i.n. delivery. The *in vivo* bio-distribution of micelles in different organs were visualized by *in vivo* imaging system (IVIS) after pulmonary delivery of MA-MC conjugated with Dylight 755 (MA-NIMc).

Figure S6 MA-ASMc are not detectable in mediastinal lymph node and spleen after pulmonary delivery. The *in vivo* bio-distribution of ASF-labeled micelles (ASMc) in different organs was tracked by flow cytometry after pulmonary administration. MA-ASMc-carrying cells were not detectable in mediastinal lymph nodes and spleens of immunized mice from 3 to 24 h after administration. Data are representative of three experiments.

Figure S7 i.n. delivery of MA-ASMc induces proliferation of adoptively-transferred DN1 T cells in the lung and spleen. Mycolic acid-specific TCR transgenic T cells (DN1) were labeled with Celltrace violet and adoptively transferred into hCD1Tg mice 1 day before immunization intranasally with MA-ASMc (*n* = 4) or V-ASMc (*n* = 3). Six days later, DN1 T cells were recovered from the lung and spleen of recipients. Representative dot plots of DN1 T cells in the lung and spleen were shown.

Figure S8 Intranasal immunization with MA-ASMc induces MA-specific T cell response in hCD1Tg CD4-deficient mice. hCD1Tg/CD4^−/−^ mice (*n* = 4) were immunized intranasally with 4 μg of MA-ASMc and sacrificed 1 week later. MA-specific, hCD1-restricted T cell response were detected in the spleen in response to re-stimulation with MA pulsed or un-pulsed hCD1Tg negative (Tg^−^) or positive (Tg^+^) MHC class II-deficient BMDCs in IFN-γ ELISPOT assay. ^*^*P* < 0.05; ^**^*P* < 0.01.

## Abbreviations

MA-Mc: mycolic acid-loaded micelle
MA-ASMc: mycolic acid-loaded acid sensitive fluorophore micelle
V- ASMc: empty/vehicle acid sensitive fluorophore micelle
MA-NIMc: mycolic acid-loaded near-infrared fluorophore micelle
V-NIMc: empty/vehicle near infrared fluorophore micelle.
