# Supplementary figures and images for: Induction of Mycobacterium Tuberculosis Lipid-Specific T Cell Responses by Pulmonary Delivery of Mycolic Acid-Loaded Polymeric Micellar Nanocarriers
Source: Front Immunol. 2018 Nov 27;9:2709. doi: 10.3389/fimmu.2018.02709 (PMC6277542; doi:10.3389/fimmu.2018.02709)

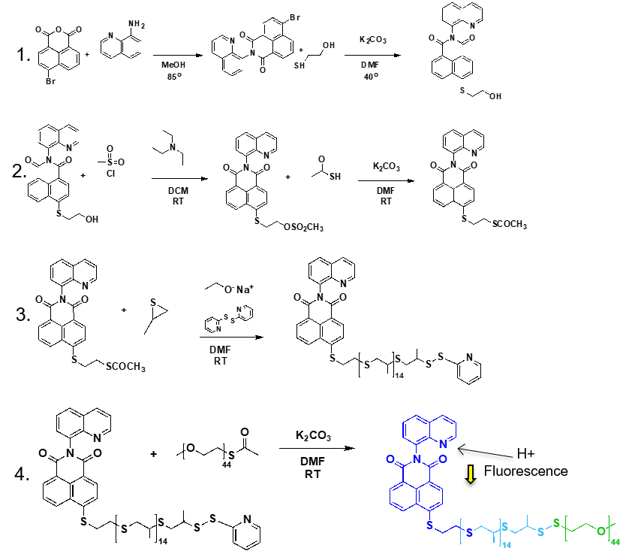

Supplement: Figure S1 — The Synthesis scheme of a poly(ethylene glycol)-bl-poly(propylene sulfide) amphiphilic copolymer tagged with a naphthalimide-derived acid sensitive fluorophore (PEG-PPS-ASF). [file Image_1.TIF]

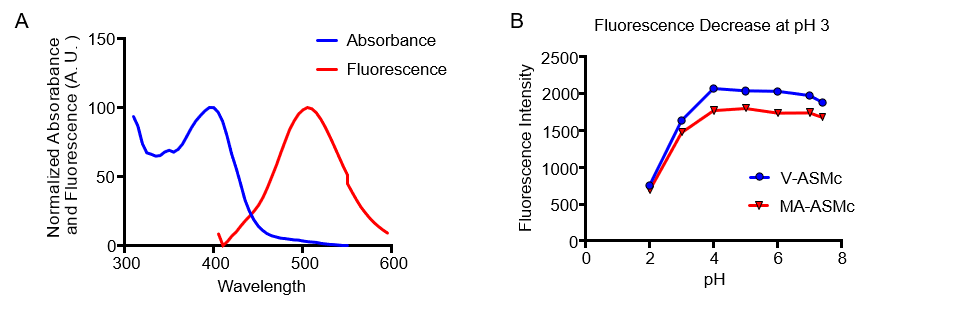

Supplement: Figure S2 — Characterization of novel acid sensitive fluorophore. (A) The fluorophore has an absorbance maximum at 395 nm and an emission maximum at 505 nm. (B) Fluorescence of empty (V-ASMc) or MA-loaded acid sensitive fluorophore tagged micelles (MA-ASMc) was measured at different pH values. Fluorescence drops after pH 3. All error bars represent SD, n = 3. [file Image_2.TIF]

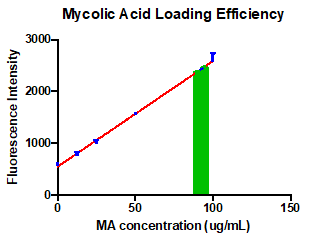

Supplement: Figure S3 — Loading efficiency of mycolic acid. Mycolic acid was conjugated to 4-bromomethyl-6,7-dimethoxycoumarin and then loaded into micelles. Fluorescence of the coumarin was measured after purification on an LH20 size exclusion column. The average loading efficiency of mycolic acid was 92 ± 3 %. Green bar represents range of loading efficiency for samples. Error bars represent SD, n = 3. [file Image_3.TIF]

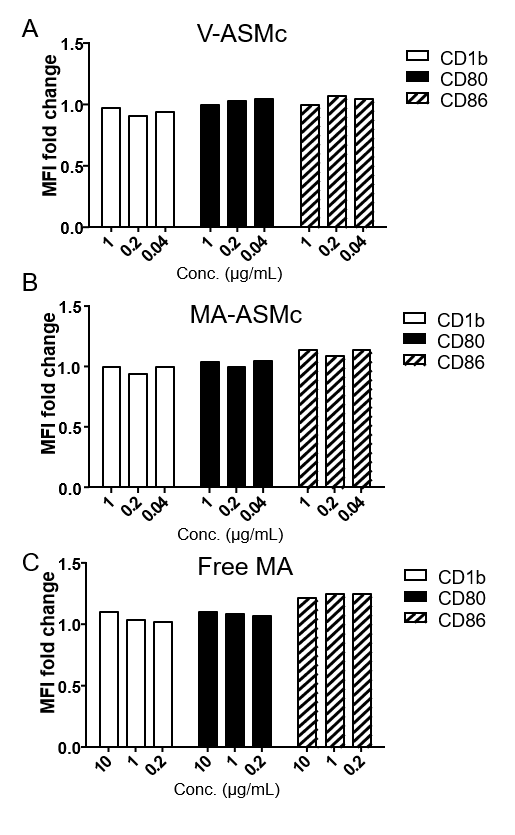

Supplement: Figure S4 — Expression changes of CD1b, CD80, and CD86 on BMDCs pulsed vs. unplused with empty micelles, MA-ASMc, and Free MA. BMDCs from hCD1Tg+ mice were differentiated in the presence of GM-CSF and IL-4 for 6 days and then pulsed or unplused with different concentration of empty micelles, MA-ASMc and Free MA for 24 h. The expression of CD1b, CD80, and CD86 on BMDCs were examined by flow cytometry. Data are expressed as fold changes of mean fluorescence intensity (MFI) of each marker on pulsed (n = 3) vs. unpulsed (n = 2) BMDCs. Data are pooled from two experiments and expressed as mean. [file Image_4.TIF]

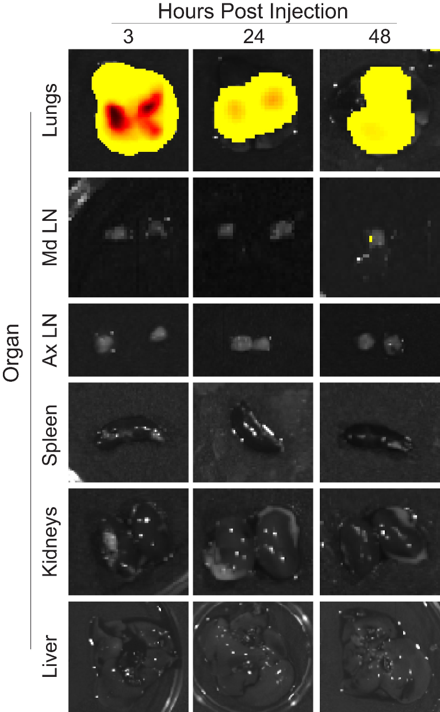

Supplement: Figure S5 — MA-NIMc are mainly retained in the lung after i.n. delivery. The in vivo bio-distribution of micelles in different organs were visualized by in vivo imaging system (IVIS) after pulmonary delivery of MA-MC conjugated with Dylight 755 (MA-NIMc). [file Image_5.TIF]

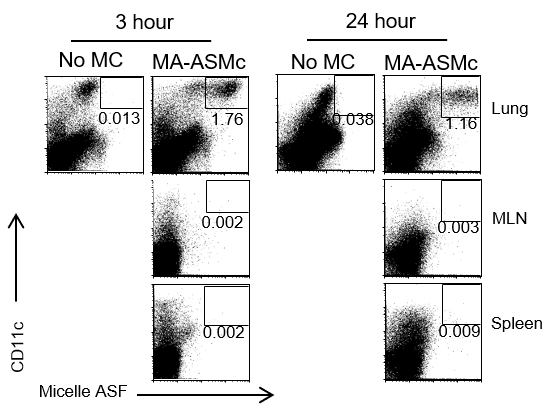

Supplement: Figure S6 — MA-ASMc are not detectable in mediastinal lymph node and spleen after pulmonary delivery. The in vivo bio-distribution of ASF-labeled micelles (ASMc) in different organs was tracked by flow cytometry after pulmonary administration. MA-ASMc-carrying cells were not detectable in mediastinal lymph nodes and spleens of immunized mice from 3 to 24 h after administration. Data are representative of three experiments. [file Image_6.TIF]

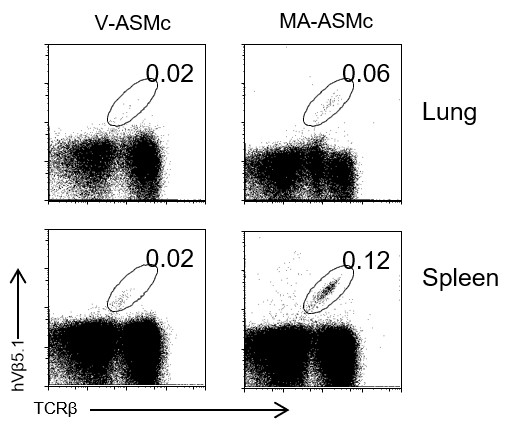

Supplement: Figure S7 — i.n. delivery of MA-ASMc induces proliferation of adoptively-transferred DN1 T cells in the lung and spleen. Mycolic acid-specific TCR transgenic T cells (DN1) were labeled with Celltrace violet and adoptively transferred into hCD1Tg mice 1 day before immunization intranasally with MA-ASMc (n = 4) or V-ASMc (n = 3). Six days later, DN1 T cells were recovered from the lung and spleen of recipients. Representative dot plots of DN1 T cells in the lung and spleen were shown. [file Image_7.TIF]

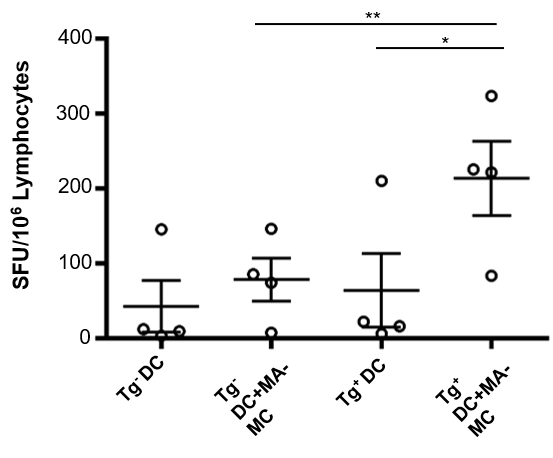

Supplement: Figure S8 — Intranasal immunization with MA-ASMc induces MA-specific T cell response in hCD1Tg CD4-deficient mice. hCD1Tg/CD4−/− mice (n = 4) were immunized intranasally with 4 μg of MA-ASMc and sacrificed 1 week later. MA-specific, hCD1-restricted T cell response were detected in the spleen in response to re-stimulation with MA pulsed or un-pulsed hCD1Tg negative (Tg−) or positive (Tg+) MHC class II-deficient BMDCs in IFN-γ ELISPOT assay. *P < 0.05; **P < 0.01. [file Image_8.TIF]
